# Emerging Targets in Clear Cell Renal Cell Carcinoma

**DOI:** 10.3390/cancers14194843

**Published:** 2022-10-04

**Authors:** Yu-Wei Chen, Brian I. Rini, Kathryn E. Beckermann

**Affiliations:** 1 Division of Hematology Oncology, Vanderbilt University Medical Center, 1211 Medical Center Drive, Nashville, TN 37232, USA; 2 Vanderbilt-Ingram Cancer Center, 2220 Pierce Ave, 777 Preston Research Building, Nashville, TN 37232, USA

**Keywords:** kidney cancer, clear cell renal cell carcinoma, RCC, trials

## Abstract

**Simple Summary:**

Immuno-Oncology (IO) based combinations are now the standard of care for frontline advanced clear cell renal cell carcinoma. Despite the unprecedented durable efficacy of such combinations, many patients do not respond to IO or will eventually develop resistance. This review summarizes the ongoing efforts in drug development in clear cell renal cell carcinoma focusing on novel targets.

**Abstract:**

The dual immune checkpoint blockade targeting CTLA-4 and PD-1 (ipilimumab/nivolumab) or the IO combinations targeting PD-1 and anti-VEGF TKIs (pembrolizumab/axitinib, nivolumab/cabozantinib, pembrolizumab/lenvatinib) have demonstrated an overall survival benefit in advanced clear cell renal cell carcinoma (ccRCC). Despite this significant improvement in clinical outcomes in the frontline setting from IO/IO or the IO/TKI combinations, there is a subset of patients of advanced ccRCC that do not respond to such combinations or will lose the initial efficacy and have disease progression. Therefore, a remarkable unmet need exists to develop new therapeutics to improve outcomes. With an enhanced understanding of ccRCC biology and its interaction with the tumor microenvironment, several new therapies are under development targeting ccRCC metabolism, cytokine-signaling, alternative immune checkpoint proteins, and novel biological pathways. In addition, microbiome products enhancing IO response, antibody–drug conjugates, and targeted radionuclides are also being investigated. This review summarizes selected emerging agents that are under development in ccRCC.

## 1. Introduction

The treatment landscape for ccRCC has evolved tremendously with the advent of immune checkpoint inhibitors (ICIs). The current standard first-line treatment for advanced ccRCC includes an immuno-oncology (IO) combination. The dual immune checkpoint blockade of ipilimumab plus nivolumab targeting cytotoxic T-lymphocytes-associated protein 4 (CTLA-4) and programmed-cell death protein 1 (PD-1), respectively, was the first and the only IO/IO combination approved by the Food and Drug Administration (FDA) based on the CheckMate 214 [1]. The other IO combination strategy involves an ICI targeting anti-PD-1 or its ligand (anti-PD-L1) plus an anti-VEGF tyrosine kinase inhibitor (TKI). There are four FDA approved combinations that belong to the IO/TKI category: pembrolizumab/axitinib [2] (KEYNOTE 426), avelumab/axitinib [3] (JAVELIN Renal 101), nivolumab/cabozantinib [4] (CheckMate 9ER), and pembrolizumab/lenvatinib [5] (CLEAR). The IO combinations brought improved overall survival benefits and unprecedented durable response in metastatic ccRCC. However, a significant subset of patients still has primary resistance to IO combinations or develop acquired resistance and eventually succumb to their disease. Therefore, there is an unmet need to develop new treatment options to improve the outcomes in ccRCC. This review will discuss how an understanding of ccRCC biology shapes current drug development in this disease and introduce emerging novel therapeutic agents that are currently under investigation.

## 2. Metabolic Inhibitors

### 2.1. Hypoxia-Inducible Factor (HIF) Inhibitor

Inactivating alterations in von Hippel-Lindau (VHL) tumor suppressor gene occurs in the majority of ccRCC tumors [6]. VHL regulates transcription factors hypoxia-inducible factor-1A (HIF-1A) and HIF-2A through ubiquitin-proteosome degradation [7]. In a hypoxic environment, the levels of HIF-1A and HIF-2A increase which upregulates erythropoietin expression and activates angiogenesis and VEGF pathways [7]. In pathogenic VHL variants, the HIF-mediated pathways are constitutively activated.

The understanding of the VHL/HIF pathway led to the development of the first-in-class HIF-2A inhibitor, belzutifan [8]. In a phase II trial consisting of RCC patients associated with VHL germline mutations (VHL disease), belzutifan showed an objective response rate of 49% (95% CI: 36–62) among 30 out of 61 patients. Following the positive results of this study, the U.S. Food and Drug Administration (FDA) approved belzutifan in patients with VHL disease who require treatment for RCC, central nervous system hemangioblastomas, or pancreatic neuroendocrine tumors not requiring immediate surgery [9].

In sporadic ccRCC, it is estimated that >90% of patients have a loss of VHL heterozygosity, and inactivating VHL mutations are found in 50–65% of cases [10] (reviewed in reference 10). The phase I LITESPARK-001 study investigated belzutifan monotherapy in solid tumors, including a cohort of pretreated ccRCC (*n* = 55, median number of prior therapies: 3). The overall response rate (ORR) was 25% and the median progression-free survival (mPFS) was 14.5 months [11]. In an updated analysis after a medium follow-up >3 years, the medium duration of response was not reached [12]. Belzutifan plus cabozantinib is being evaluated in the treatment-naïve (cohort 1) and previously treated advanced ccRCC (cohort 2). The preliminary results of cohort 2 showed an ORR of 22% (9 partial responses among 41 patients) and a disease control rate of 92.7%. Of note, 53% had prior first-line therapy and 45% had prior second-line therapy. The mPFS was 16.8 months and the duration of response was not reached [13]. Most common treatment-related adverse event (TRAE) included anemia (76%; grade 3: 11.3%), fatigue (68%; grade 3: 11.3%), hand-foot syndrome (52.8%), diarrhea (45%), and hypertension (43%; grade 3: 22.4%). There was no grade 4 TRAEs or treatment-related death.

Ongoing trials are investigating belzutifan in the first or later lines of therapy for patients with advanced ccRCC as monotherapy or in various combinations (Table 1). Of note, a phase III randomized-controlled study investigating belzutifan versus everolimus in the second/third line setting in ccRCC (NCT04195750) has completed enrollment. For first-line treatment, a phase III triplet study investigating the combination of belzutifan with pembrolizumab/lenvatinib or quavonlimab (anti-CTLA4) with pembrolizumab/lenvatinib vs. pembrolizumab/lenvatinb [14] (NCT04736706) is ongoing. Belzutifan is also being investigated in combination with pembrolizumab (versus pembrolizumab monotherapy) in the adjuvant setting after nephrectomy [15] (NCT05239728).

Other HIF-2A targeted agents are under development. ARO-HIF2 is a synthetic double-stranded RNA interference (RNAi) that is given by intravenous infusion. The initial results of the ongoing phase I ARO-HIF21001 (NCT04169711) demonstrated decreased HIF-2A mRNA expression after treatment, and the safety profile was favorable. Disease control was observed in 7 out of 23 patients (30%) [16]. Another oral HIF-2A small molecule inhibitor, NKT2152, is being investigated in a phase 1/2 dose-escalation/expansion study (NCT05119335).

### 2.2. Glutaminase Inhibitor

Glutamine has been reported to be a vital nutrient source in many cancer cells, including ccRCC [17,18,19,20]. The glutamine transporters, which transport glutamine from the blood into the cells, have higher expression in ccRCC [21] compared to normal renal cells. The constitutively activated HIF pathway in ccRCC also shifts the cancer cells to utilize more glutamine than glucose to fuel the tricarboxylic acid (TCA) cycle [18,21]. Glutamine is imported into mitochondria and is converted to glutamate by the enzyme glutaminase [22] in the TCA cycle. Telaglenastat (CB-839) is a first-in-class glutaminase inhibitor and has shown antitumor activity and favorable safety profile in early phase studies in ccRCC as monotherapy [23] or in combinations with everolimus or cabozantinib [24]. The phase II double-blinded placebo-controlled ENTRATA trial (NCT03163667) compared telaglenastat plus everolimus vs. everolimus in a cohort of heavily treated ccRCC (median of three prior lines of systemic therapy) [25]: telaglenastat plus everolimus showed an improved mPFS (3.8 months vs. 1.9 months, HR: 0.64, 95% CI: 0.34–1.2, *p*-value: 0.079) which met the trial primary endpoint (one-sided alpha < 0.2). However, in another similarly designed phase II CANTANA trial [26] (NCT03428217), telaglenastat plus cabozantinib failed to show improved efficacy compared to cabozantinib alone (mPFS: 9.2 months vs. 9.3 months, *p*-value: 0.65). The contrasting results of these trials emphasize the need to better understand the involved biological mechanisms and pathways to select partnering agents with telaglenastat in ccRCC. Further correlative data from both trials hopefully can bring more insights to guide future trial design [25,26] using glutaminase inhibitors.

### 2.3. Adenosine Inhibition

The accumulation of adenosine and its interaction with the adenosine 2A receptors (A2AR) on immune cells in the tumor microenvironment (TME) has been shown to restrict the anti-tumor activity of cytotoxic T cells and natural killer (NK) cells [27,28,29]. In addition, adenosine also augments the immunosuppressive activity of regulatory T cells (Treg) and myeloid-derived suppressor cells (MDSC) [29,30,31]. Targeting the adenosine-mediated pathway can potentially reverse the resistance to immune checkpoint inhibitors in preclinical studies [32,33]. Ciforadenant (CPI-444) is a small molecule that competes with adenosine to bind the A2AR and inhibits the signaling of the pathway [34]. In a first-in-human phase I study, ciforadenant was given as a monotherapy or in combination with anti-PD-L1 atezolizumab in an advanced RCC cohort of 68 patients, of which 72% were resistant or refractory to prior anti-PD1/PD-L1 therapy [35]. The safety profile was acceptable in both the ciforadenant monotherapy and combination arms. Partial response was observed in 1 of 33 (3%) patients treated with ciforadenant monotherapy and 4 of 35 (11%) patients treated with ciforadenant/atezolizumab. The disease control rate at 6 months was 39% in the combination vs. 17% in the monotherapy in this patient population with a median of three prior lines of therapy. The survival endpoints also favored the combination (mPFS: 5.8 months vs. 4.1 months; OS at 25 months: 90% vs. 69%). The efficacy suggested that the combination had higher anti-tumor activity than the ciforadenant monotherapy, although this trial was not designed to compare the two arms. A correlative study revealed the efficacy of ciforadenant was associated with CD8^+^ T-cell infiltration and diversification of the T-cell receptor repertoire; this trial also proposed an adenosine-related gene signature as a predictive biomarker [35]. There is a planned phase II trial of ciforadenant in combination with ipilimumab and nivolumab in the first-line treatment of advanced RCC through the Kidney Cancer Research Consortium [36].

Blocking the formation of adenosine is another potential mechanism to inhibit adenosine-mediated immunosuppression in the TME. Adenosine triphosphate (ATP) serves as the energy source in cells. In the extracellular space, ATP is also a signaling molecule [37]. Extracellularly, ATP is metabolized to adenosine monophosphate and then adenosine through CD39 and CD73, respectively [38]. CD39 and CD73 are both ectonucleotidases expressed on the cell membrane [37]. Preclinical research suggested that CD73 inhibition could enhance the antitumor activity of ICIs [39]. BMS-986179 is an anti-CD73 antibody and was investigated as a monotherapy or in combination with nivolumab in a phase I/IIa study consisting of pre-treated advanced solid tumor patients. Among 59 patients treated with BMS-986179 with/without nivolumab, 7 patients had a partial response, and 10 patients had stable disease. The combination safety profile was similar to nivolumab monotherapy [40].

## 3. Cytokines

### 3.1. Interleukin-2

A small subset of advanced RCC patients treated with high-dose (HD) interleukin-2 (IL-2) can achieve complete response and have durable long-term remission [41]. However, the overall response rate is low, and the significant muti-organ adverse events, including cardiac toxicities of HD IL-2, require inpatient delivery and close monitoring. Therefore, the use of HD IL-2 is limited at experienced centers. The interest in HD IL-2 has reemerged in the ICI era. A single institution trial combined HD IL-2 with pembrolizumab in advanced RCC patients without prior PD-1/PD-L1 therapy showed a promising response among 19 of 25 patients (79%) [42]. There are ongoing trials investigating HD IL-2 with anti-PD-1 antibodies in the frontline setting or after disease progression on PD-1/PD-L1 antibodies (Table 2).

Efforts have been made to improve the anti-tumor activity of IL-2-based therapy and to reduce the associated toxicities. Nemvaleukin alfa (ALKS-4230) is a fusion protein of permuted IL-2 to the extracellular domain of IL-2 receptor α (IL-2Rα). It is designed to simulate the intermediate but not the high-affinity IL-2R, leading the IL-2 fusion protein to preferentially stimulate effector T cells [43] and not stimulate Tregs [43]. There is an ongoing phase I/II study assessing nemvaleukin alfa monotherapy or in combination with pembrolizumab in second-line solid tumors (ARTISTRY-1, NCT02799095). Part B of the protocol included an advanced RCC-focused cohort: 4 of 21 evaluable patients had an objective response and 11 had a stable disease when nemvaleukin was given as monotherapy [44]. Part C of this trial is investigating nemvaleukin with pembrolizumab in solid tumors. The preliminary results showed an ORR of 16.1% (22/137) and a disease control rate of 59.9% in this heavily pretreated solid tumor cohort (1 to 9 prior lines of therapy, including ICI) [45].

Bempegaldesleukin (NKTR-214) is a modified pegylated (PEG) IL-2R agonist which preferentially binds to IL-2Rβ over IL-2Rα [46]. Compared to unmodified conventional IL-2, bempegaldesleukin leads to more CD8+ T cell and NK cell activation/expansion and less Treg expansion and is, thus, hypothesized to have increased anti-tumor activity and reduced toxicities [46]. The FDA granted breakthrough therapy designation to bempegaldesleukin in August 2019 and several phase III clinical trials were opened in multiple tumor types. In March 2022, the first analysis of the phase III PIVOT IO-001 trial comparing bempegaldesleukin/nivolumab vs. nivolumab in untreated metastatic melanoma showed no improved clinical benefit of this combination [47]. In April 2022, negative findings of this combination were also reported in the phase III PIVOT-09 trial in advanced ccRCC (comparator: TKI monotherapy) and the phase II PIVOT-10 trial in cisplatin-ineligible urothelial cancer. Future development of bempegaldesleukin in combination with nivolumab has been halted [48]. Perhaps the PEG modification to decrease Treg activity from bempegaldesleukin ultimately also decreased the needed CD8 T cell effector function leading to ultimately negative trials across several tumor types.

### 3.2. Interleukin-15

Interleukin 15 (IL-15) has similar immunostimulatory properties as IL-2 on NK and T effector cells aiding in activation and expansion but has less interaction with Tregs [49]. The half-life of human IL-15 is short (<1 h), limiting the clinical implications [50]. The IL-15 superagonist, SOT101 (formerly SO-C101), is a fusion molecule of human IL-15 that is linked to IL-15Rα [49] and has increased stability and activity delivered subcutaneously [50,51]. A phase I study assessing SOT101 as monotherapy and in combination with pembrolizumab in solid tumors, including ccRCC, is ongoing (NCT04234113) (Table 2).

### 3.3. Interleukin-27 Inhibitor

IL-27 consists of two subunits: IL27p28 and Epstein-Barr virus-induced gene 3 (EBI3). IL-27 binds to its receptor, which is composed of glycoprotein 130 (gp130) and IL-27RA. IL-27 has an immunoregulatory effect. It can upregulate the inhibitory immune checkpoint receptors and downregulate proinflammatory cytokines [52,53]. The Cancer Genome Atlas (TCGA) dataset revealed higher transcript expression of EBI3, IL-27RA, and IL27p28 in ccRCC tumors compared to normal kidney tissue. In addition, higher transcript expression of EBI3, IL27p28, and IL-27RA was associated with worse overall survival in ccRCC [54].

Inhibiting IL-27-mediated signaling theoretically can augment antitumor activity. SRF388 is a first-in-class anti-IL-27p28 antibody that blocks the interaction between IL-27 and IL-27RA [54]. SRF388 is being investigated in a phase I dose-escalation and expansion study, including a cohort of ccRCC. SRF388 showed a favorable safety profile as monotherapy or in combination with pembrolizumab. The preliminary results revealed encouraging monotherapy activity of SRF388 in the ccRCC (of the 10 evaluable ccRCC patients, one had confirmed partial response) and met the criteria to expand the cohort for further investigation [55] (NCT04374877) (Table 2).

## 4. Targeting New Immune Checkpoints Proteins

### 4.1. LAG3 Inhibitor

Lymphocyte-activation gene 3 (LAG3) is an inhibitory receptor that is expressed on activated T cells, Tregs, and NK cells [56]. LAG3 binds to its major ligand, the major histocompatibility complex-II (MHC-II), on the antigen-presenting cells, which prevents its interaction with the T cell receptor. This leads to the inhibition of T-cell receptor signaling and downregulates T-cell proliferation and cytokine release [57,58]. LAG-3 and PD-L1 are often found to be co-expressed on tumor-infiltrating T cells, and dual blockade of LAG-3 and PD-1 has shown synergistic anti-tumor efficacy in preclinical studies [59,60].

Relatlimab is the first-in-class anti-LAG-3 antibody. In a randomized double-blinded phase III study in frontline advanced melanoma (RELATIVITY-047), the combination of relatlimab with nivolumab showed a superior PFS compared to nivolumab monotherapy (10.1 months vs. 4.6 months, HR: 0.75, 95% CI: 0.62–0.92, *p*-value: 0.006) [61] and subsequently received FDA approval [62]. The overall survival data has not yet matured. An ongoing randomized phase II trial investigating several nivolumab-based combinations vs. ipilimumab/nivolumab in advanced RCC includes one arm of relatlimab with nivolumab (NCT02996110) (Table 2). Relatlimab/nivolumab is also being investigated in the neoadjuvant setting of ccRCC in a phase II trial (NCT05148546) (Table 2).

Ieramilimab (LAG525) is another LAG-3 inhibitor under investigation. In a completed phase I/II study of Ieramilimab with or without the anti-PD-1 spartalizumab (PDR001) in 255 advanced solid tumor patients, including RCC, ieramilimab was well tolerated and showed modest antitumor activity in the combination arm (*n* = 121, 3 had a complete response, 10 had a partial response, and 35 had stable disease) including patients pre-treated with anti-PD-1/PD-L1 [63].

### 4.2. TIGIT Inhibitor

T cell immunoreceptor with immunoglobulin and ITIM domains (TIGIT) is a receptor of the Ig family and is expressed on activated CD8^+^ or CD4^+^ T cells, Tregs, and NK cells [64]. There are three ligands for TIGIT expressed on antigen-presenting cells and cancer cells: CD155, CD112, and CD113. Compared to CD112 and CD113, TIGIT has the highest affinity with CD155. The interaction of TIGIT and its ligands causes altered functions of the antigen-presenting cells and decreased cytokine release leading to reduced T cell activation [65]. TIGIT has been reported to be expressed on exhausted CD8^+^ T cells in RCC [66]. Preclinical studies have demonstrated antitumor activities with the combination of TIGIT blockade and anti-PD-1/anti-PD-L1 inhibition [67,68].

Tiragolumab is an anti-TIGIT monoclonal antibody. Early phase studies showed favorable tolerability of Tiragolumab monotherapy and in combination with atezolizumab. In a randomized phase II placebo-controlled study of PD-L1 positive non-small cell lung cancer in the frontline setting, the combination of tiragolumab/atezolizumab showed improved mPFS (5.4 months vs. 3.6 months, HR: 0.57, 95% CI: 0.37–0.90, *p*-value: 0.015) and objective response rate (31% vs. 16%, *p*-value: 0.03) compared to placebo/atezolizumab [69]. A phase II study investigating tiragolumab with atezolizumab in advanced solid tumors, including RCC, is ongoing (NCT03977467) (Table 2).

### 4.3. ILT2/ILT4 Inhibitors

Ig-like transcript 2 (ILT2) and 4 (ILT4) are immune-inhibitory receptors that are expressed in the NK cells and T cells [70]. The human leukocyte antigen-G (HLA-G) is the ligand for ILT2/ILT4 that has increased expression in most cancers, including 50% of RCC [71]. HLA-G has been reported to be associated with inflammation and poor prognosis [72].

NGM707 is a dual antagonist for ILT2/ILT4. A preclinical study has shown that NGM707 stimulates the activation of myeloid/lymphoid cells and reprograms suppressive myeloid cells [73]. There is an ongoing phase I/II dose escalation/expansion study of NGM707 with/without pembrolizumab in solid tumors, including RCC (NCT04913337) (Table 2).

### 4.4. ILT3 Inhibitor

Myeloid-derived suppressor cells (MDSCs) have been shown to impair antitumor activity and promote immunosuppression leading to a pro-tumorigenic environment [74]. Ig-like transcript 3 (ILT3, also called LILRB4) is a receptor expressed on MDSC shown to promote immunosuppressive function [75]. A phase I/Ib study of ILT3 inhibitor (NGM831) with/without pembrolizumab has been launched in advanced solid tumors (NCT05215574) (Table 2).

### 4.5. TREM2 Inhibitor

Tumor-associated macrophages (TAMs) are immunosuppressive cells in the TME and have been shown to contribute to T-cell dysfunction/exhaustion, resistance to anti-PD1/PDL1 therapy, and associated with worse clinical outcomes [76,77].

TREM2 is a transmembrane protein that is highly expressed on TAMs [78]. Compared to normal renal tissue, TREM2-expressing macrophages were found to be higher in abundance in the ccRCC tumors. In addition, high TREM-2 expressing macrophages were associated with a higher risk of recurrence [79]. PY314 is a monoclonal antibody against TREM2 and has shown efficacy as monotherapy and in combination with an anti-PD1 agent in the preclinical study [80]. A phase Ia/Ib investigating PY314 with/without pembrolizumab in advanced solid tumors is ongoing (NCT04691375) (Table 2).

### 4.6. OX40 Agonist

Full T cell activation requires both T cell receptor signaling and activation of its co-stimulatory receptors [81]. OX40 (CD134) is a co-stimulatory molecule that is mainly expressed on activated T cells [81]. The binding of OX40 with its ligand (OX40L), which is primarily expressed on antigen-presenting cells, promotes T cell expansion and prolongs their survival [82]. Preclinical studies have revealed synergistic antitumor activity combining anti-PD-1 and OX40 agonists [83,84].

A phase I dose-escalation study investigating OX40 agonist PF-04518600 in solid tumors showed a favorable toxicity profile and preliminary anti-tumor activity [85]. However, in a subsequent phase II study in advanced ccRCC after progression of PD-1/PD-L1 inhibition, axitinib with PF-04518600 did not improve clinical outcomes compared to axitinib alone (median PFS: 13.1 vs. 8.5 months, HR: 0.85, 95% CI: 0.45–1.60, *p*-value: 0.61) [86]. In another phase I/IIa study investigating OX40 agonist BMS-986178 in solid tumors including RCC, BMS-986178 was well tolerated either as monotherapy or in combination with nivolumab and/or ipilimumab but did not show clear efficacy [87].

INBRX-106 is a novel hexavalent OX40 agonist under development. Preclinical studies showed it outperformed bivalent antibodies in costimulatory capacity and anti-tumor activity [88]. INBRX-106 is being investigated in a phase I dose-escalation study with/without pembrolizumab in advanced solid tumors (NCT04198766) (Table 2).

## 5. Novel Mechanisms

### 5.1. Batiraxcept (AVB-S6-500)

AXL is a member of the TAM (Tyro3, Axl, Mer) receptor tyrosine kinase family, which has a high affinity for its ligand growth arrest-specific protein 6 (Gas6) [89]. AXL is highly expressed in many cancer cells. The Gas6/AXL signaling promotes cellular invasion/migration and has been shown to be associated with treatment resistance and poor clinical outcomes [90,91,92]. In ccRCC, the constitutively activated HIF pathway leads to increased expression of AXL [93], and the Gas6/AXL pathway has been associated with treatment resistance of anti-VEGF TKIs [94]. In addition, Gas6/AXL also fosters immunosuppressive TME by promoting the infiltrations of macrophages, MDSCs, and monocytes and decreasing the infiltrations of T effector cells [95].

Batiraxcept (AVB-S6-500) is a first-in-class fusion protein consisting of the extracellular domain of the AXL receptor fused to a human immunoglobulin G1 Fc domain [96]. Batiraxcept has a 200-fold higher affinity to Gas6 than the AXL receptor [97] and can effectively neutralize the Gas6 protein and inhibit AXL signaling. In a preclinical study of the ovarian cancer mouse model, batiraxcept improved tumor response to chemotherapy and PARP inhibitor [98]. Batiraxcept is being investigated in a phase Ib/II trial in ccRCC in combination with cabozantinib in 2nd line therapy, in combination with cabozantinib/nivolumab in frontline treatment, and lastly as monotherapy in the refractory setting (NCT04300140) (Table 3). The preliminary results of the phase Ib portion of the trial showed favorable safety/tolerability in combination with cabozantinib (no dose-limiting toxicity observed) [99,100] and ORR of 46% (12 partial responses out of 26 patients) [101]. The trial is enrolling the expansion cohorts in this and other line settings [100].

### 5.2. Adavosertib (AZD1775)

The cell cycle consists of 4 phases: G1, S, G2, and M, and the cyclin-dependent kinases (CDK) are responsible for the regulation of the cell cycle and DNA replication [102]. In response to DNA damage, the WEE1 kinase, which is a serine-threonine kinase [103], suppresses the CDK1/2 activity by phosphorylation. This results in cell cycle pause at the intra-S or G2/M checkpoint, allowing DNA damage repair to occur prior to mitosis and avoiding aberrant DNA-induced apoptosis [104]. This process maintains the integrity of the cell’s genome [105]. Inhibition of WEE1 increases the activity of CDK1, which results in cells passing through the G2-M checkpoint unregulated and subsequently leads to the accumulation of unrepaired DNA damage and cell deaths [106]. Inhibition of WEE1 also increases the activity of CDK2, which results in aberrant DNA replication [107]. WEE1 inhibition with adavosertib (AZD1775) has been shown to increase the sensitivity of chemotherapy and radiotherapy in preclinical and early phase studies [103]. Of note, in a preclinical study in SETD2-deficient cancers, WEE1 inhibition with adavosertib was shown to have antitumor activity in vitro and in vivo [108]. In fact, ccRCC is the most common SETD2-inactivated cancers [109]. It has been observed that 10% of primary ccRCC tumors and 30% of metastatic ccRCC tumors have SETD2 mutations, suggesting its role in driving tumor progression [6,110]. A phase II trial investigating adavosertib in SETD-2 deficient cancers, including a cohort for advanced ccRCC, is ongoing (NCT03284385) (Table 3).

### 5.3. DS-6000a

The successful development of HER-2 targeted antibody-drug conjugate (ADC) trastuzumab deruxtecan (T-DXd) in advanced breast cancer [111] has led to the further investigation of DXd-based ADC in ccRCC. DS-6000a is a DXd-based ADC that targets the human cadherin 6 (CDH6), which is a transmembrane protein overexpressed in ovarian cancer and RCC [112]. The DXd-based ADC is internalized after binding to its target cells, and the linker is degraded intracellularly by lysosomal enzymes to release deruxtecan which causes DNA damage and apoptotic cell death. Because of the bystander effect, ADC not only kills the cells it binds to but also has anti-tumor activity against neighboring cells. DS-6000a is currently being investigated in a phase I trial that includes an expansion cohort of ccRCC (NCT04707248) (Table 3). The interim results showed acceptable tolerability and early signs of antitumor activity (2 had a partial response and 9 had stable disease among 15 evaluable patients) in heavily pretreated RCC [113].

## 6. Tyrosine Kinase Inhibitors

### 6.1. Sitravatinib

Sitravatinib is a multi-targeted tyrosine kinase inhibitor targeting TAM (Tyro3, Axl, Mer) receptors, VEGFR, c-MET, and c-KIT. Preclinical data showed that sitravatinib could reduce immune-suppressive myeloid cells in the TME and shift the TME to a more proinflammatory phenotype [114]. In a phase I/II study, sitravatinib was combined with nivolumab in advanced immunotherapy-naïve ccRCC that progressed after 1 or 2 prior anti-angiogenic regimens (*n* = 42). After a median follow-up of 18.7 months, the sitravatinib/nivolumab combination demonstrated an ORR of 35.7% and a median progression-free survival of 11.7 months [115]. Sitravatinib/nivolumab was also evaluated in a phase II neoadjuvant study in ccRCC patients who underwent curative nephrectomy [116]. Sitravatinib was given for the first two weeks and nivolumab was added after. The total treatment was 6–8 weeks prior to nephrectomy. There were 17 patients evaluable for efficacy and 2 patients had partial response [116].

Sitravatinib/nivolumab is currently being investigated in a single-arm phase II study of advanced ccRCC after the progression of prior immunotherapy (NCT04904302) (Table 3). Another ongoing phase I study investigates sitravatinib/ipilimumab/nivolumab in treatment-naive advanced ccRCC [117] (NCT04518046) (Table 3).

### 6.2. XL092

XL092 is a novel oral tyrosine kinase inhibitor that targets VEGFR, AXL, MER, and MET kinases similar to cabozantinib with a shorter half-life. There are two ongoing phase I studies investigating XL092 as monotherapy or in combination as doublets or triplets when combined with other IO agents (NCT03845166; NCT05176483) (Table 3). Each of these studies includes an expansion cohort focusing on advanced ccRCC [118,119].

## 7. Live Microbiome Product

### CBM588

Several studies have shown that the composition of the gut microbiome modulates the treatment response of ICIs in several cancers [120,121,122]. There are many species proposed to have such an effect, including *Bifidobacterium* spp. In a preclinical study, oral administration of *Bifidobacterium* improves the anti-tumor activity of the anti-PD-L1 antibody against melanoma [123]. In a retrospective study consisting of nivolumab or ipilimumab/nivolumab-treated mRCC, patients’ gut microbiome enriched in *Bifidobacterium* spp. was associated with improved clinical response. The early studies have led to the development of a phase I trial investigating CBM588 given with ipilimumab/nivolumab in first-line mRCC [124]. CBM588 is an orally administered live *Clostridium butyricum* product that has been shown to increase the abundance of gut *Bifidobacterium* spp. [125]. In this study, patients with International Metastatic RCC Database Consortium (IMDC) intermediate and poor risk were randomized to ipilimumab/nivolumab with (*n* = 19; poor risk: 11%) or without (*n* = 10; poor risk 30%) CBM588. After a medium follow-up of 12.2 months, patients in the CBM588/ipilimumab/nivolumab arm had superior mPFS (12.7 vs. 2.5 months, HR: 0.15, 95% CI: 0.05–0.47, *p*-value < 0.001). Among responders, patients who received CBM588 had an interval increase in gut *Bifidobacterium* spp. from baseline to week 12 in stool samples; such an increase was not observed among non-responders. The results should be interpreted with caution given the small sample size [124] and future studies are warranted to confirm this finding. There is an ongoing phase I trial investigating CBM588 with an IO/TKI combination of nivolumab/cabozantinib in ccRCC (NCT05122546) (Table 3).

## 8. Targeted Radionuclide Therapy

### 8.1. Lu-177-Girentuximab

The successful developments of Lutetium-177 (Lu-177) dotatate and Lu-177 prostate-specific membrane antigen (PSMA)-617 in advanced neuroendocrine tumor [126] and castration-resistant prostate cancer [127], respectively, have brought targeted radionuclide therapy to daily oncology practice. The selective binding of the radioligand to the specific marker expressed by a given tumor is the key concept to maximizing therapeutic efficacy and minimizing toxicities of the radionuclide [128]. In ccRCC, a potential tumor target is carbonic anhydrase IX (CAIX), which is ubiquitously expressed in ccRCC (both primary and metastatic tumors) but not in normal kidney tissue [129]. Girentuximab is a monoclonal antibody that targets CAIX [130]. Lu-177-girentuximab was previously investigated in a small single-arm phase II study [131] consisting of progressive ccRCC before the ICI era. Among the 14 enrolled patients, eight patients had stable disease after the first cycle and one had a partial response. Grade 3–4 myelotoxicities were observed in most patients. Six patients received the second cycle and five patients had continued stable disease. None of the patients received further treatment due to prolonged thrombocytopenia [131]. Currently, Lu-177-girentuximab is being investigated as a combination with nivolumab in previously treated ccRCC (NCT05239533) (Table 3). Of note, the eligibility criteria require at least one evaluable metastatic lesion on zirconium-89 (89Zr)-girentuximab PET/CT [132]. The diagnostic value of 89Zr-girentuximab PET/CT in ccRCC is also being evaluated in a phase III trial (NCT03849118).

### 8.2. Lu-177-EB-PSMA-617

PSMA is a transmembrane glycoprotein encoded by the folate hydrolase 1 gene (FOLH1) and is highly expressed in prostate adenocarcinoma [133]. However, PSMA is a misnomer and it is not specific to prostate cancer. In fact, PSMA is also expressed in the neovasculature of many solid tumors [134]. As a result of upregulated HIF and VEGF pathways, ccRCC is characterized by substantial neovasculature with high expression of PSMA. Several studies have investigated the diagnostic utility of PSMA PET/CT in kidney cancer [134,135]. The current data suggest that PSMA PET/CT may aid in detecting metastatic RCC lesions but has a limited role in staging the primary tumors, given the uptake of PSMA by normal renal parenchyma. Given the success of Lu-177-PSMA-617 in prostate cancer, using the same rationale targeting PSMA in non-prostate tumors may be feasible. There is an ongoing trial using Lu-177-EB-PSMA-617, which conjugates a truncated Evans blue (EB) molecule to PSMA-617, to investigate its use in detection and therapeutic benefits in RCC [136] (NCT05170555) (Table 3).

## 9. Novel Trial Design

### 9.1. Triplet Studies

While IO/IO and IO/TKI doublets have become the standard frontline treatments for advanced ccRCC, the field has continued to evolve and investigate the efficacy of triplet combinations. COSMIC-313(NCT03937219) (Table 4) is a phase III randomized placebo-controlled trial comparing the triplet regimen of cabozantinib/ipilimumab/nivolumab vs. ipilimumab/nivolumab in IMDC intermediate and high-risk advanced ccRCC. This is the first study to use a standard of care IO combination as the control. Cabozantinib has been shown to have an immunomodulatory effect [137] and the rationale of this triplet is to increase the clinical response of ipilimumab/nivolumab. The results of COSMIC 313 were presented at the European Society for Medical Oncology (ESMO) Congress 2022 meeting. The triplet of cabozantinib/ipilimumab/nivolumab showed superior PFS over ipilimumab/nivolumab (mPFS: not reached vs. 11.3 months; HR: 0.73, 95% CI: 0.57–0.94, *p*-value: 0.013) and met the trial primary endpoint. The secondary endpoint of OS is not mature yet. The ORR was numerically higher in the triplet (43%, 95% CI: 37.2–49.2) than ipilimumab/nivolumab (36%, 95% CI: 30.1–41.8). Grade 3/4 TRAEs were 73% in the triplet versus 41% in the control. Of note, 45% of patients in the triplet arm had treatment discontinuation, while it was 24% in the control [138].

Other triplets under investigation include the previously mentioned phase III study combining the IO/TKI backbone of pembrolizumab/lenvatinib with belzutifan or the anti-CTLA4 agent quavonlimab (NCT04736706) (Table 4). Moreover, a phase 1b/2 triplet umbrella study includes the same triplets and also additional combinations of favezelimab (anti-LAG-3) with pembrolizumab/lenvatinib, and vibostolimab (anti-TIGIT) with pembrolizumab/belzutifan (NCT 04626479) (Table 4).

### 9.2. Adaptive Trial Design-PDIGREE

In contrast to triplet combinations upfront, the PDIGREE trial (NCT03793166) (Table 4) uses an adaptive trial design that intensifies treatment sequentially based on the first scan obtained after completing ipilimumab/nivolumab induction [139]. At the 3-month response assessment, patients with progressive disease are assigned to cabozantinib 60 mg daily. Patients with complete responses will be assigned to nivolumab maintenance. Patients with partial response or stable disease are randomized to the cabozantinib (40 mg daily)/nivolumab vs. nivolumab monotherapy. The PDIGREE trial will help identify the timing and patient subsets that will benefit from adding TKI to the standard nivolumab maintenance after ipilimumab/nivolumab induction.

### 9.3. Biomarker Driven Design- OPTIC RCC

The current standard first-line treatment for advanced ccRCC involves either an IO/TKI or IO/IO combination. However, there is no level-1 evidence to guide the treating physicians to choose one over the other category. A correlative study of the phase III IMmotion 151 study used the RNA-sequencing from 823 ccRCC tumors and characterized them into seven biologically distinct clusters. Based on the biology of the clusters and their response to immunotherapy, the phase II OPtimal Treatment by Invoking biologic Clusters in Renal Cell Carcinoma (OPTIC RCC) trial (NCT05361720) (Table 4) adopts a biomarker-driven design to assign protocol treatment. The hypothesis is that gene expression clusters will characterize a patient’s tumor biology to select frontline treatment with either cabozantinib/nivolumab or the IO/IO combination of ipilimumab/nivolumab. The primary endpoint is ORR and will compare efficacy to historical control in unselected patients.

## 10. Conclusions

**Figure 1:**
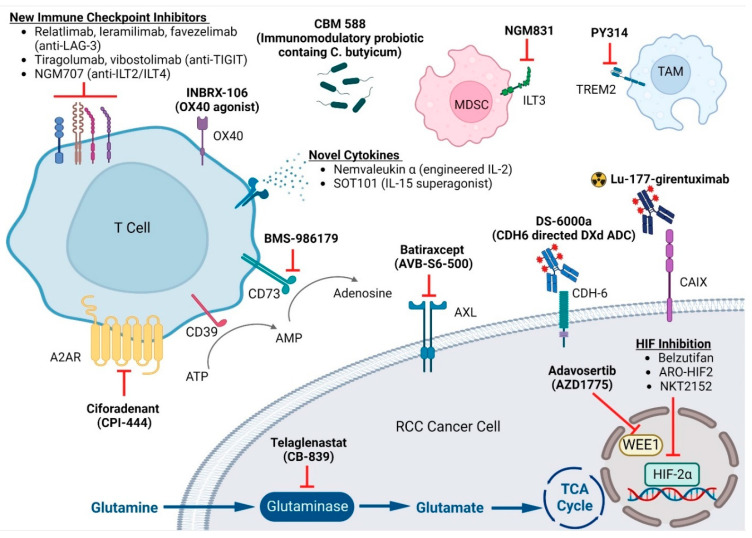
Emerging Targets in Clear Cell Renal Cell Carcinoma. This figure is created with BioRender.com. LAG-3: Lymphocyte-activation gene 3; TIGIT: T cell immunoreceptor with Ig and ITIM domains; ILT: Ig-like transcript; MDSC: Myeloid-derived suppressor cell; TAM: tumor-associated macrophage; A2AR: adenosine 2A receptors; ATP: adenosine triphosphate; AMP: adenosine monophosphate; CDH6: human cadherin 6; CAIX: carbonic anhydrase IX; TCA: tricarboxylic acid; HIF: hypoxia-inducible factor.

While IO-based combinations have dramatically improved the care of patients with metastatic ccRCC, there are still patients who do not respond to frontline IO-based combinations or, after initial benefit, ultimately develop progressive disease on this treatment. Thus, a remarkable unmet need remains to develop novel treatment options in this field. This review article summarized novel and encouraging therapeutics that are currently in development (Figure 1).

With an improved understanding of the ccRCC biology, tumor metabolism, and tumor microenvironment, we foresee the treatment paradigm in ccRCC would enter an era that adopts biomarkers to select treatment options for this heterogenous disease in the near future.

## Figures and Tables

**Table 1:** Ongoing clinical trials of HIF inhibitors.

Treatment	Setting	Phase	Primary Endpoints	Trial Number
Belzutifan (dose-escalation study)	2L+ advanced ccRCC	I	AEs; treatment discontinue/interruption/modification rate; DLT rate	NCT04846920
Belzutifan + Cabozantinib	Cohort 1: 1L advanced ccRCC Cohort 2: prior immunotherapy and no more than two prior treatments	II	ORR	NCT03634540
Pembrolizumab +/− belzutifan	Adjuvant therapy in ccRCC post nephrectomy	III	DFS	NCT05239728
Belzutifan + Pembrolizumab/Lenvatinib or Quavonlimab + Pembrolizumab/Lenvatinib Comparator: Pembrolizuamb/Lenvatinib	1L advanced ccRCC	III	PFS/OS	NCT04736706
Belzutifan+ Lenvatinib Comparator: Cabozantinib	2L+ (after anti-PD-1/PD-L1)	III	PFS/OS	NCT04586231
Belzutifan Comparator: everolimus	2L/3L (after anti-PD-1/PD-L1 and an anti-VEGF TKI in sequence or in combination);	III	PFS/OS	NCT04195750
ARO-HIF2 (Dose-finding study)	2L+ (after anti-VEGF TKI and immune checkpoint inhibitor therapy)	1b	AEs	NCT04169711
NKT2152 (Dose-escalation and expansion trial)	2L+ (after anti-PD-1/PD-L1 and/or an anti-VEGF agent)	1/2	Phase I: Number of participants with DLT; RP2D Phase II: ORR	NCT05119335

AEs: adverse events; DLT: dose-limiting toxicity; ORR: overall response rate; DFS: disease-free survival; 1L: first line; PFS: progression-free survival; OS: overall survival; 2L+: second-line or later; 2L: second line. RP2D: recommended phase 2 dose.

**Table 2:** Ongoing clinical trials targeting cytokines and new immune checkpoints.

Treatment	Setting	Phase	Primary Endpoints	Trial Number
**Cytokines**				
IL-2 with nivolumab	2L+ (after anti-PD-1/PD-L1)	II	ORR	NCT03991130
IL-2 with pembrolizumab	2L+ (after anti-PD-1/PD-L1)	II	ORR	NCT05155033
IL-2 with pembrolizumab	Any line	II	AEs	NCT03260504
Nemvaleukin alfa (engineered IL-2) monotherapy +/−pembrolizumab	After the progression of standard of treatment	I/II	DLT, AEs; ORR	NCT02799095
SOT101 (IL-15 superagonist) monotherapy +/−pembrolizumab	After the progression of standard of treatment	I	DLT, AEs,	NCT04234113
SRF388 monotherapy SRF388 with pembrolizumab	Advanced solid tumors that have progressed after standard of treatment	I/Ib	DLT, ORR, AEs	NCT04374877
**Immune Checkpoint Inhibitors**				
LAG-3 inhibitor: Relatlimab/nivolumab Comparator: Ipilimumab/nivolumab	Advanced RCC	II	ORR DOR PFS	NCT02996110
LAG-3 inhibitor: Relatlimab/nivolumab (arm C)	Neoadjuvant	II	Pathologic response rate	NCT05148546
TIGIT inhibitor: Tiragolumab with atezolizumab (arm B)	Advanced solid tumors that have progressed after standard of treatment	II	ORR	NCT03977467
ILT2/ILT4 inhibitor: NGM 707 with/without pembrolizumab	Advanced solid tumors that have progressed after standard of treatment	I/II	Phase I: DLT, AE Phase II: ORR, DOR, PFS, OS	NCT04913337
ILT3 inhibitor: NGM831 with/without pembrolizumab	Advanced solid tumors that have progressed after standard of treatment	I/Ib	DLT, AEs Lab abnormalities	NCT05215574
TREM2 inhibitor: PY314 with/without pembrolizumab	Advanced solid tumors that have progressed after standard of treatment	Ia/Ib	AE, DLT	NCT04691375
OX40 agonist: INBRX-106 with/without pembrolizumab	Advanced solid tumors that have progressed after standard of treatment	I	AE MTD, RP2D	NCT04198766

2L+: second-line or later; ORR: overall response rate; AEs: adverse events; DLT: dose-limiting toxicity; DOR: duration of response; PFS: progression-free survival; OS: overall survival.

**Table 3:** Ongoing clinical trials targeting novel mechanisms.

Treatment	Setting	Phase	Primary Endpoints	Trial Number
**AXL inhibitor**				
Batiraxcept (AVB-S6-500) Batiraxcept + Cabozantinib Batiraxcept + Cabozantinib/Nivolumab	2L+ 2L 1L	Ib/II	AEs, RP2D, ORR, DOR, PFS, OS	NCT04300140
**WEE1 inhibitor**				
Adavosertib (AZD1775)	2L+ in SETD2-deficient ccRCC (cohort B)	II	ORR	NCT03284385
**Antibody-drug conjugate**				
DS-6000a	ccRCC: 2L+	I	DLT AEs	NCT04707248
**New multitargeted TKIs**				
Sitravatinib/nivolumab	2L+ after progression of immunotherapy	II	ORR DCR	NCT04904302
Sitravatinib/nivolumab/ipilimumab	1L	I/Ib	AEs	NCT04518046
XL092 XL092 + Atezolizumab XL092 + Avelumab	Dose-escalation cohort: advanced solid tumors Expansion Cohort A: 2L+ ccRCC	I	MTD, RD ORR PFS	NCT03845166
XL092 XL092 + Nivolumab XL092 + Ipilimumab/Nivolumab XL092+ Nivolumab/Bempegaldesleukin	Dose-escalation cohort: advanced solid tumors Expansion Cohort 1: 1L ccRCC Expansion Cohort 2: 2L+ ccRCC	Ib	AEs ORR PFS	NCT05176483
**Microbiome product**				
CBM588+ Nivolumab/Cabozantinib Comparator: nivolumab/cabozantinib	advanced ccRCC, no prior immunotherapy	I	Change in Bifidobacterium composition of stool at week 12	NCT05122546
**Targeted Radionuclide Therapy**				
Lu-177-girentuximab with Nivolumab	2+ (at least one prior treatment with anti-PD1/PD-L1)	II	MTD, ORR	NCT05239533
Lu-177-EB-PSMA-617	Treated or untreated RCC	n/a	Diagnostic value of 68Ga-PSMA PET/CT for RCC	NCT05170555

2L+: second-line or later; AEs: adverse events; RP2D: recommended phase 2 dose; ORR: overall response rate; DOR: duration of response; PFS: progression-free survival; OS: overall survival. DLT: dose-limiting toxicity; DCR: disease control rate; 1L: first line. MTD: maximum tolerated dose; RD: recommended dose.

**Table 4:** Ongoing clinical trials using triplets or novel trial designs.

Treatment	Setting	Phase	Primary Endpoints	Trial Number
cabozantinib/ipilimumab/nivolumab vs. Ipilimumab/nivolumab	1L IMDC intermediate/high-risk ccRCC	III	PFS	NCT03937219
Belzutifan + Pembrolizumab/Lenvatinib or Quavonlimab + Pembrolizumab/Lenvatinib Comparator: Pembrolizuamb/Lenvatinib	1L advanced ccRCC	III	PFS/OS	NCT04736706
Quavonlimab Pembrolizumab/Lenvatinib or Favezelimab (anti-LAG-3) + Pembrolizumab/Lenvatinib or Belzutifan+ Pembrolizuamb/Lenvatinib or Vibostolimab (anti-TIGIT) + Pembrolizuamb/belzutifan	1L advanced ccRCC	1b/II (Umbrella study)	DLT, AEs, discontinuation rate; ORR	NCT 04626479
**Adaptive Trial Design-PDIGREE** After completion of ipilimumab/nivolumab induction, patients with PD will receive cabozantinib 60 mg daily; CR patients will receive nivolumab. Patients with PR/SD will be randomized to cabozantinib/nivolumab vs. nivolumab	1L IMDC intermediate/high risk advanced ccRCC	III	OS	NCT03793166
**Biomarker-Driven Design-OPTIC RCC** Treatment assigned based on RCC tumor gene expression profile: cabozantinib/nivolumab or ipilimumab/nivolumab	1L advanced ccRCC	II	ORR	NCT05361720

1L: first line; PFS: progression-free survival; OS: overall survival; DLT: dose-limiting toxicity; AEs: adverse events; ORR: overall response rate.
